# Equity in access to health care among asylum seekers in Germany: evidence from an exploratory population-based cross-sectional study

**DOI:** 10.1186/s12913-015-1156-x

**Published:** 2015-11-09

**Authors:** Kayvan Bozorgmehr, Christine Schneider, Stefanie Joos

**Affiliations:** Department of General Practice & Health Services Research, University Hospital Heidelberg, Voßstr.2, Geb. 37, 69115 Heidelberg, Germany; Institute of General Practice and Interprofessional Care, University of Tuebingen, Tuebingen, Germany

**Keywords:** Asylum-seekers, Refugees, Access to health care, Subjective social status, Education, Inequality, Equity, Migration, Unmet need, Socioeconomic status

## Abstract

**Background:**

Research on inequities in access to health care among asylum-seekers has focused on disparities *between* asylum-seekers and resident populations, but little attention has been paid to potential inequities in access to care *within* the group of asylum-seekers. We aimed to analyse the principles of horizontal equity (i.e., equal access for equal need irrespective of socioeconomic status, SES) and vertical equity (higher allocation of resources to those with higher need) among asylum-seekers in Germany.

**Methods:**

We performed a secondary exploratory analysis on cross-sectional data obtained from a population-based questionnaire survey among all asylum-seekers (aged 18 or above) registered in three administrative districts in Germany during the three-month study period (*N* = 1017). Data were collected on health care access (health care utilisation of four types of services and unmet medical need), health care need (approximated by sex, age and self-rated health status), and SES (highest educational attainment and subjective social status, SSS). We calculated odds ratios and 95 % confidence intervals (CI) in multiple logistic regression models to analyse associations between SES indicators and access to health care under control of need.

**Results:**

We contacted 60.4 % (614) of the total asylum-seekers population, of which 25.4 % (*N* = 156) participated in the study. Educational attainment showed no significant effect on health care access in crude models, but was positively associated with utilisation of psychotherapists and hospital admissions in adjusted models. Higher SSS was positively associated with health care utilisation of all types of services. The odds of hospitals admissions for asylum-seekers in the medium and highest SSS category were 3.18 times [1.06, 9.59] and 1.6 times [0.49, 5.23] the odds of those in the lowest SSS category. After controlling for need variables none of the SES indicators were significantly associated with measures of access to care, but a positive association remained, indicating higher utilisation of health care among asylum-seekers with higher SES. Age, sex or general health status were the only significant predictors of health care utilisation in fully adjusted models. The adjusted odds of reporting unmet medical needs among asylum-seekers with “fair/bad/very bad” health status were 2.16 times [0.84, 5.59] the odds of those with “good/very good” health status.

**Conclusion:**

Our findings revealed that utilisation of health services among asylum-seekers is associated with higher need (vertical equity met). Horizontal equity was met with respect to educational attainment for most outcomes, but a social gradient in health care utilisation was observed across SSS. Further confirmatory research is needed, especially on potential inequities in unmet medical need and on measurements of SES among asylum-seekers.

**Electronic supplementary material:**

The online version of this article (doi:10.1186/s12913-015-1156-x) contains supplementary material, which is available to authorized users.

## Background

Unprecedented levels of forced migrants seeking asylum in other countries [1] challenge the provision of high-quality care in line with the values of European health systems such as equity in access to health care [2]. In 2013, the European Union (EU) received almost half a million asylum-seekers - a 32 % increase on numbers from 2012 [3].

Access to health care is initially restricted for asylum-seekers in many European countries, despite the principles of universality and equity [4]. Germany, the country receiving the highest absolute number of asylum applications worldwide [1], has set up legal restrictions on access to health care for asylum-seekers. These have been in place since the 1990s. Entitlements to medical care are detailed in the Asylum-Seekers’ Benefits Act (AsylbLG), a national law which restricts access to health care. Services covered include emergency care, care for acute and painful conditions, care during pregnancy and child birth, vaccinations and other “indicated preventive measures” (AsylbLG sections 4 and 6) [5].

These legal restrictions have been criticised by scholars as “third class medicine” [6] because they create inequities in access to care *between asylum-seekers and the resident population*. Less academic attention has been paid to the potential equity effects of bureaucratic regulations, which have the potential to create inequities in access to health care *within the group of asylum-seekers*. Any type of health care utilisation (except for emergencies) for asylum-seekers in Germany is conditional on the receipt of a health care voucher, which has to be granted by the welfare agency after personal request in all but the smallest Federal States. Additional care (according to AsylbLG section 6) may be granted upon formal request if the measures are deemed to be essential to preserve health – an issue which is subject to the latitude of judgement of local authorities and may depend on the ability of asylum-seekers to express, verbalise and negotiate their needs on an interpersonal level with administrative staff. Further detailed information on legal entitlements, regulations and barriers to health care for asylum-seekers in Germany can be found in other publications [5].

A solid body of international evidence points to high needs for health care among asylum-seekers related to mental health [7, 8], infectious diseases [9, 10] and chronic diseases [10, 11]. Most of this evidence is generated from comparisons with resident populations or with non-refugee migrant populations in receiving countries.

However, asylum-seekers are a heterogeneous migrant group with respect to their health care needs and their exposure to pre- and peri-migration health risks. Individual resources that may affect health status and health care seeking behaviour in the host country include cultural and religious background, education and socioeconomic status (SES) [12]. It is well known that SES - traditionally composed of education, income and profession – is inversely associated with health care need (i.e., morbidity) and positively associated with access to health care [13] in most societies.

However, it is not known whether SES affects access to health care *within* the heterogeneous group of asylum-seekers – particularly in countries which have established “parallel systems” of care as is the case in Germany. Furthermore, it is unknown if the restrictions and regulations of the “parallel system” [5] adequately allocate health care resources according to need. We thus set out to analyse whether the principles of horizontal equity in health care (i.e., equal access for equal need irrespective of SES [2]) and vertical equity in health care (i.e., the appropriate unequal treatment of unequals [14] or the higher allocation of resources according to higher need [15]) are met *within* a population of asylum-seekers in Germany.

We primarily aimed to analyse whether SES is associated with health care access among asylum-seekers after controlling for differences in morbidity, age and sex. The secondary aim was to assess if need was appropriately associated with access to health care after controlling for SES.

## Methods

### Design

This study is based on a secondary analysis of data obtained from a population-based cross-sectional study with a full-census approach in a convenience sample of three administrative districts in Baden-Württemberg, one of the largest Federal States with 44 administrative districts in the South of Germany. The analysis is explorative in the sense that no sample size calculation was performed in advance due to a lack of studies with similar scope among asylum-seeking populations. This means that effect estimates are judged upon regarding their direction and magnitude rather than upon their statistical significance (or non-significance).

### Participants

Asylum-seekers were considered individuals who have applied for recognition as refugee in Germany and are awaiting a decision on their application (*AsylVfG section 55*), are “tolerated” (*AufenthaltG section 60*) or hold a permit on humanitarian grounds (*AufenthaltG section 25*). Asylum-seekers aged 18 or above were eligible to participate in the study.

### Data collection and recruitment

The study protocol was approved by the ethical committee of the Medical Faculty of the University of Heidelberg prior to onset of the study (Ethical approval nr: S-261/2014). Data were collected between October 2014 and February 2015 on the occasion of monthly payments of welfare benefits to asylum-seekers either in the accommodation centre or in the Welfare Agency, where all registered asylum-seekers are obliged to be personally on-site. In a full census approach, all asylum-seekers meeting the inclusion criteria were invited to participate. They were informed by the research team in written and oral form about the voluntary nature of participation, anonymity and confidentiality of data, emphasizing that participation would neither influence the health care situation nor the asylum procedure or residence status. Data were collected on health care access, morbidity, and SES using a questionnaire with mainly standardized instruments in seven languages (German, English, French, Arabic, Persian, Serbian, Russian) tailored to the languages most frequently spoken among registered asylum-seekers. Details on the translation process are provided in Additional file 1. Demographic data on non-responders and reasons for declining participation were documented to perform a non-responder analysis.

### Measurement of access (dependent variables)

Access to health care as an outcome is a multi-layered concept to be best approximated by measuring health service utilization and unmet need [16–18]. To quantify utilisation participants were asked whether they had used any of the following medical services during the past 12 months: (1) Any physician (in-or outpatient); (2) General practitioner (GP); (3) Psychotherapist; (4) Hospital inpatient. Unmet medical need was recorded if participants indicated that at least once in the past 12 months they had felt a need for medical care but did not receive it. Each item was used as a binary dependent variable in different models.

### Measurement of SES (independent variables)

Capturing and comparing SES among asylum-seekers in the traditional “objective” sense is challenging as it requires measurement of SES indicators (education, income, profession) in a fashion that is applicable to both a broad array of countries (e.g., .standardised measurement of education and professions) and to the legal particularities entailed by the asylum-seeking process. At the time of data collection for this study, asylum-seekers did not receive working permits during the first 9 months of their stay in the country. Further regulations limiting “real” access to the labour market during the first 15 months after receipt of a working permit rendered questions for income inapplicable and difficult to operationalise [19].

Since the impact of social standing in society on health goes beyond the effects of objective SES and includes the effects of subjective positioning into the social hierarchy that societies create [20, 21], we decided to capture SES using a two-pronged strategy:

Firstly, measurement of the highest educational attainment as “objective” SES indicator by the question “What is the highest education you received?” with the response options “None at all; Primary school; Secondary school; Religious school; Tertiary/University”. The option “religious school” was collapsed with “Secondary school”, so that the highest educational attainment was used as an ordinal variable with four categories (none/primary/secondary/tertiary education).

Secondly, we captured the participants’ subjective social status (SSS) in Germany as comprehensive measure of SES by asking participants to put themselves on a 10-rung social ladder modelled after the MacArthur Scale [20, 22]. The SSS index was grouped into three groups: Low (1–4), Medium (5–6), High (7–10) [23].

Both measures of SES were used as independent variables in separate models. In models that contained both measures jointly, we used SSS as independent and educational attainment as control variable.

### Measurement of need (independent variables)

Following common approaches [14], we used self-rated general health status, quantified on a five-point Likert scale and dichotomized into ‚Good’ (very good/good) and ‚Bad’ (fair/bad/very bad), to approximate morbidity and need for health care among asylum-seekers. Further need variables were age (continuous) and sex, which we included hypothesising that higher age and female sex indicate higher health care needs.

### Control variables

The place of residence (district 1, 2 or 3) was included as a covariable to assess potential differences in access that are explained through geographic characteristics. Furthermore, a language variable with three categories (‘German’, ‘English’ and ‘Other language’) was generated and included in the multiple regression analysis if the language proved to be significantly associated with health care access in the bivariate models.

### Statistical analysis

We calculated sex-stratified absolute and relative frequencies for categorical variables, and means, medians and standard deviations (SD) as well as the interquartile range (IQR) for continuous variables. To assess the relationship between dependent, independent and control variables, we designed causal diagrams to visualise the potential relationship between the variables and guide the development of statistical models (Additional file 1). The number of variables was restricted in favour of a higher model power.

We calculated unadjusted Odds Ratios (OR) with 95 % confidence intervals [CI] in simple logistic regression models for each pair of dependent and independent variables. Multiple logistic regression analysis was performed to investigate the adjusted association between dependent and independent variables as well as the joint explanatory effect of the predictor variables on access to health care. Variation inflation factors (VIFs) were calculated to assess multicollinearity among covariables and all VIFs of included variables ranged between 1 and 2.

We built three models for each combination of SES indicators: model 1 contained educational attainment as main independent variable, model 2 SSS as main independent variable, and model 3 both educational attainment and SSS (whereas the first was used as control variable). Separate models were built for each of the five dependent variables: (A) Physician, (B) GP, (C) Psychotherapist, (D) Hospital, (E) Unmet medical need, so that in total 15 models were analysed (Model 1A–E, Model 2A–E, Model 3A–E). A stepwise inclusion of the need and control variables was performed as illustrated in Additional file 2.

For the analysis of horizontal equity, the final models describe the association between SES and access to care under control of sex, age and morbidity. The analysis of vertical equity includes the SES adjusted association of need variables on access to care. Goodness of fit was assessed using the Bayesian information criterion (BIC). All statistical tests were calculated on a significance level of alpha = 0.05 in an exploratory manner and analyses were carried out with STATA 12.1.

### Missing data and non-responder

Missing data were treated as missing at random and a complete case analysis was performed. We performed a non-responder analysis to assess potential differences in sex and language between responding and non-responding AS.

## Results

### Descriptive results

Of 1017 asylum-seekers registered in the three administrative districts during the study period, 614 (60.4 %) could be contacted and 156 asylum-seekers from 22 countries participated in the study, yielding a response rate of 25.4 %. In total, 15.3 % of the asylum-seekers population in the region participated with more men (64.7 %) than women (22.4 %) among the participants (Table 1). Median duration of stay in Germany was 16 months (IQR 6; 39) among the total population, 18.5 months among men (IQR 7; 41) and 7 months among women (IQR 3; 27). The response rate was highest for English speaking asylum-seekers, followed by German, Serbian, Persian and Arabic (Table 1).

**Table 1:** Descriptive characteristics of participating asylum seekers (*N* = 156)

		Male	Female	Gender not specified	Total	Missings per item
Sociodemographic data		Freq. (col %)				Freq. (% of N)
Place of residence	County 1	31 (30.7)	18 (51.4)	12 (60)	61 (39.1)	
	County 2	29 (28.7)	9 (25.7)	3 (15)	41 (26.3)	
	County 3	41 (40.6)	8 (22.9)	5 (25)	54 (34.6)	
	N (%)	101 (100)	35 (100)	20 (100)	156 (100)	0 (0.0)
Country of origin	Iran	10 (12.8)	3 (10.3)	0 (0)	13 (12)	
	Pakistan	9 (11.5)	3 (10.3)	0 (0)	12 (11.1)	
	Gambia	9 (11.5)	1 (3.4)	1 (100)	11 (10.2)	
	Macedonia	8 (10.3)	3 (10.3)	0 (0)	11 (10.2)	
	Afghanistan	9 (11.5)	0 (0)	0 (0)	9 (8.3)	
	Iraq	5 (6.4)	4 (13.3)	0 (0)	9 (8.3)	
	Serbia	6 (7.7)	3 (10.3)	0 (0)	9 (8.3)	
	Other	22 (28.2)	12 (41.4)	0 (0)	34 (31.5)	
	N (%)	78 (100)	29 (100)	1 (100)	108 (100)	48 (30.7)
Language	Arabic	8 (7.9)	3 (8.6)	4 (20)	15 (9.6)	
	German	16 (15.8)	14 (40)	3 (15)	33 (21.2)	
	English	34 (33.7)	6 (17.1)	8 (40)	48 (30.8)	
	French	3 (3)	1 (2.9)	1 (5)	5 (3.2)	
	Persian	18 (17.8)	4 (11.4)	2 (10)	24 (15.4)	
	Russian	5 (5)	2 (5.7)	1 (5)	8 (5.1)	
	Serbian	17 (16.8)	5 (14.3)	1 (5)	23 (14.7)	
	N (%)	101 (100)	35 (100)	20 (100)	156 (100)	0 (0.0)
Age group	18–29	48 (53.9)	14 (53.8)	0 (0)	62 (53.9)	
	30–39	24 (27)	4 (15.4)	0 (0)	28 (24.4)	
	40–49	13 (14.6)	4 (15.4)	0 (0)	17 (14.8)	
	>50	4 (4.5)	4 (15.4)	0 (0)	8 (7)	
	Mean (SD)	31.0 (9.5)	34.1 (13.3)	n.a.	31.73 (10.5)	
	N (%)	89 (100)	26 (100)	0 (0)	115 (100)	41 (26.3)
Highest degree of education	None	14 (14.6)	7 (20)	0 (0)	21 (15.7)	
	Primary school	24 (25)	12 (34.3)	0 (0)	36 (26.9)	
	Secondary school	30 (31.2)	6 (17.1)	3 (100)	39 (29.1)	
	University	28 (29.2)	10 (28.6)	0 (0)	38 (28.4)	
	N (%)	96 (100)	35 (100)	3 (100)	134 (100)	22 (14.1)
Subjective social status	Lower	30 (38.0)	6 (23.1)	0 (0)	36 (33.6)	
	Middle	26 (32.9)	9 (34.6)	1 (50)	36 (33.6)	
	High	23 (29.1)	11 (42.3)	1 (50)	35 (32.7)	
	N (%)	79 (100)	26 (100)	2 (100)	107 (100)	49 (31.4)
Self-reported health status						
General state of health	Very bad	4 (4.1)	0 (0)	0 (0)	4 (2.7)	
	Bad	20 (20.4)	6 (17.6)	3 (17.65)	29 (19.5)	
	Fair	25 (25.5)	8 (23.5)	5 (29.41)	38 (25.5)	
	Good	30 (30.6)	14 (41.2)	2 (11.8)	46 (30.9)	
	Very good	19 (19.4)	6 (17.6)	7 (41.2)	32 (21.5)	
	N (%)	98 (100)	34 (100)	17 (100)	149 (100)	7 (4.5)
Utilization of health services^a^						
Physician (any type)	Yes	79 (78.2)	29 (82.9)	11 (55)	119 (76.3)	
General Practitioner	Yes	65 (64.4)	24 (68.6)	9 (45)	98 (62.8)	
Specialist	Yes	21 (20.8)	11 (31.4)	3 (15)	35 (22.4)	
	N (%)	101 (100)	35 (100)	20 (100)	156 (100)	0 (0.0)
Psychotherapist	Yes	16 (15.8)	6 (17.1)	2 (10)	24 (15.5)	
	N (%)	101 (100)	35 (100)	20 (100)	155 (100)	1 (0.6)
Hospital	Yes	22 (22)	11 (32.4)	6 (30)	39 (25.3)	
	N (%)	100 (100)	34 (100)	20 (100)	154 (100)	2 (1.2)
Unmet needs^a^						
Experienced unmet need	Yes	42 (44.7)	11 (31.4)	10 (50)	63 (43.5)	11 (7.1)
	Total	94 (100)	35 (100)	20 (100)	145 (100)	

^a^All data on utilisation of health care services/health care access refer to the past 12 months

More than 50 % of respondents were aged below 30 years (53.9 %) and reported a degree from secondary school or a university degree as highest educational attainment (57.5 %). The responses across the three SSS categories were equally distributed among the total population. In the low SSS category, the proportion of men was 15 percentage-points higher compared to women, while in the high SSS category the proportion of men was 13 percentage-points lower compared to women (Table 1).

About 76 % of the respondents had seen any physician in the last 12 months. Utilisation of GPs was reported most frequently (62.8 %) and of psychotherapists least frequently (15.5 %). The prevalence of unmet medical need among respondents was 43.5 %, and about 13 percentage-points higher among male than among women. The prevalence of ‘Bad’ (fair/bad/very bad) general health status was 47.7 % among the total population, 50 % among men and 41.1 % among women (Table 1).

There were no significant sex differences between participants and non-responders (*p* = 0.542). The main reason for non-response was lack of a common language between researchers and asylum-seekers. Further details on non-responders are provided in Additional file 3.

### Unadjusted simple regression estimates

The unadjusted simple regression estimates obtained from bivariate models showed statistically significant positive associations between age, “Bad” health status and utilisation of any type of physician (in- or outpatient) (Table 2) and GPs (Table 3). Utilisation of psychotherapists (Table 4) and unmet medical need (Table 6) were significantly and positively associated with “Bad” health status.

**Table 2:** Crude and adjusted regression estimates for the association between utilisation of physicians (in-and outpatient) and SES or need variables among asylum seekers

Explanatory variables		Bivariate models	Multiple regression models (adjusted for sex, age and general health)		
OR [95 % CI]			Model I	Model II	Model III
			Education	SSS	Education + SSS
Education	None/Primary *(Ref.)*	1 *(Ref.)*	1 *(Ref.)*	-	1 *(Ref.)*
	Secondary	0.82 [0.29,2.32]	1.25 [0.25,6.14]	-	1.46 [0.19,11.47]
	Tertiary	0.8 [0.28,2.25]	0.83 [0.19,3.69]	-	0.45 [0.08,2.65]
SSS^a^	Lower *(Ref.)*	1 *(Ref.)*	-	1 *(Ref.)*	1 *(Ref.)*
	Middle	1.6 [0.41,6.23]	-	4.09 [0.57,29.42]	5.55 [0.65,41.0]
	High	0.58 [0.18,1.84]	-	1.45 [0.28,7.43]	1.37 [0.24,7.37]
Sex	Male *(Ref.)*	1 *(Ref.)*	1 *(Ref.)*	1 *(Ref.)*	1 *(Ref.)*
	Female	1.35 [0.50,3.65]	1.59 [0.33,7.74]	2.29 [0.34,15.44]	2.11 [0.32,13.79]
Language	German *(Ref.)*	1 *(Ref.)*	1 *(Ref.)*	1 *(Ref.)*	1 *(Ref.)*
	English	**0.34 [0.12,0.98]**	0.61 [0.11,3.42]	1.53 [0.23,10.42]	1.74 [0.21,14.73]
	Others^c^	1.17 [0.4,3.43]	2.11 [0.38,11.73]	5.63 [0.78,40.5]	7.34 [0.86,62.73]
Age (yrs)		**1.16 [1.06,1.27]**	**1.14 [1.02,1.28]**	**1.22 [1.03,1.45]**	**1.23 [1.03,1.47]**
General health status^b^	“Good” *(Ref.)*	1 *(Ref.)*	1 *(Ref.)*	1 *(Ref.)*	1 *(Ref.)*
	“Bad”	**3.44 [1.48,8.00]**	**5.75 [1.32,25.03]**	**6.91 [1.43,33.5]**	4.8 [0.9,25.5]
BIC		-	109.33	93.1	100.31
N		-	108	94	93

*OR* indicates the odds ratio compared to the reference group, *CI* indicates 95 % confidence interval, *Pseudo R* ^*2*^ squared for logistic regression, *N* absolute frequency of participating persons. Variations in N result from missing in single items. All data on utilisation of health care services refer to the past 12 months. ^a^SSS: Subjective social status in Germany on a scale from 1 to 10. Lower: 1–4 points. Middle: 5–6 points. High: 7–10 points. ^b^“How is your health in general?“ “Bad” = fair/bad/very bad. “Good” = good/very good. ^c^Other languages: Arabic, French, Persian, Russian, Serbian. Bold figures: Reflect ORs which are smaller/larger than 1 with a confidence level of 95 %

**Table 3:** Crude and adjusted regression estimates for the association between utilisation of general practitioners and SES or need variables among asylum seekers

Explanatory variables		Bivariate models	Multiple regression models (adjusted for sex, age and general health)		
OR [95 % CI]			Model I	Model II	Modell III
			Education	SSS	Education + SSS
Education	None/Primary *(Ref.)*	1 *(Ref.)*	1 *(Ref.)*	-	1 *(Ref.)*
	Secondary	0.85 [0.35,2.04]	1.64 [0.49,5.53]	-	1.40 [0.32,6.04]
	Tertiary	0.58 [0.25,1.38]	0.55 [0.18,1.72]	-	0.37 [0.10,1.38]
SSS^a^	Lower *(Ref.)*	1 *(Ref.)*	-	1 *(Ref.)*	1 (Ref.)
	Middle	1.45 [0.55,3.84]	-	2.11 [0.62,7.22]	2.74 [0.74,10.18]
	High	1.22 [0.46,3.21]	-	2.46 [0.67,9.04]	2.46 [0.64,9.47]
Sex	Male *(Ref.)*	1 *(Ref.)*	1 *(Ref.)*	1 *(Ref.)*	1 *(Ref.)*
	Female	1.21 [0.53,2.75]	1.92 [0.58,6.34]	2.04 [0.48,8.61]	2.06 [0.469.14]
Language	German *(Ref.)*	1 *(Ref.)*	1 *(Ref.)*	1 *(Ref.)*	1 *(Ref.)*
	English	**0.34 [0.13,0.86]**	0.33 [0.08,1.28]	0.29 [0.06,1.26]	0.32 [0.06,1.66]
	Others^c^	1.12 [0.46,2.74]	1.28 [0.36,4.54]	1.45 [0.36,5.9]	2.06 [0.44,9.6]
Age (yrs)		**1.07 [1.02,1.13]**	1.05 [0.99,1.1]	**1.06 [1.00,1.13]**	**1.07 [1.00,1.15]**
General health status^b^	“Good” *(Ref.)*	1 *(Ref.)*	1 *(Ref.)*	1 *(Ref.)*	1 *(Ref.)*
	“Bad”	**2.08 [1.05,4.11]**	1.45 [0.56,3.75]	1.35 [0.48,3.8]	0.85 [0.27,2.72]
BIC		-	153.37	133.93	138.1
N		-	108	94	93

*OR* indicates the odds ratio compared to the reference group, *CI* indicates 95 % confidence interval, *Pseudo R* ^*2*^ squared for logistic regression, *N* absolute frequency of participating persons. Variations in N result from missing in single items. All data on utilisation of health care services refer to the past 12 months. ^a^SSS: Subjective social status in Germany on a scale from 1 to 10. Lower: 1–4 points. Middle: 5–6 points. High: 7–10 points. ^b^“How is your health in general?“ “Bad” = fair/bad/very bad. “Good” = good/very good. ^c^Other languages: Arabic, French, Persian, Russian, Serbian. Bold figures: Reflect ORs which are smaller/larger than 1 with a confidence level of 95 %

**Table 4:** Crude and adjusted regression estimates for the association between utilisation of psychotherapists and SES or need variables among asylum seekers

Explanatory variables		Bivariate models	Multiple regression models (adjusted for sex, age and general health)		
OR [95 % CI]			Model I	Model II	Model III
			Education	SSS	Education + SSS
Education	None/Primary *(Ref.)*	1 *(Ref.)*	1 *(Ref.)*	-	1 *(Ref.)*
	Secondary	1.21 [0.43,3.41]	2.07 [0.52,8.16]	-	2.07 [0.46,9.30]
	Tertiary	0.71 [0.22,2.28]	1.41 [0.32,6.26]	-	1.47 [0.31,6.97]
SSS^a^	Lower *(Ref.)*	1 *(Ref.)*	-	1 *(Ref.)*	1 *(Ref.)*
	Middle	1 [0.31,3.21]	-	1.13 [0.29,4.36]	1.1 [0.27,4.44]
	High	1.23 [0.39,3.85]	-	1.55 [0.39,6.25]	1.44 [0.34,5.98]
Sex	Male *(Ref.)*	1 *(Ref.)*	1 *(Ref.)*	1 *(Ref.)*	1 *(Ref.)*
	Female	1.1 [0.39,3.07]	1.03 [0.26,4.05]	1.29 [0.31,5.27]	1.46 [0.33,6.38]
Language	German *(Ref.)*	1 *(Ref.)*	1 *(Ref.)*	1 *(Ref.)*	1 *(Ref.)*
	English	**0.18 [0.04,0.72]**	0.19 [0.03,1.11]	0.44 [0.08,2.54]	0.3 [0.04,2.01]
	Others^c^	0.52 [0.19,1.38]	0.53 [0.14,1.94]	1.03 [0.26,4.01]	0.78 [0.18,3.39]
Age (yrs)		0.96 [0.91,1.02]	0.94 [0.88,1.01]	0.95 [0.89,1.02]	0.95 [0.88,1.02]
General health status^b^	“Good” *(Ref.)*	1 *(Ref.)*	1 *(Ref.)*	1 *(Ref.)*	1 *(Ref.)*
	“Bad”	**3.44 [1.26,9.38]**	**4.41 [1.29,15.14]**	**4.60 [1.27,16.73]**	**5.10 [1.28,20.38]**
BIC		-	125.65	118.02	125.38
N		-	108	94	93

*OR* indicates the odds ratio compared to the reference group, *CI* indicates 95 % confidence interval, *Pseudo R* ^*2*^ squared for logistic regression, *N* absolute frequency of participating persons. Variations in N result from missing in single items. All data on utilisation of health care services refer to the past 12 months. ^a^SSS: Subjective social status in Germany on a scale from 1 to 10. Lower: 1–4 points. Middle: 5–6 points. High: 7–10 points. ^b^“How is your health in general?” “Bad” = fair/bad/very bad. “Good” = good/very good. ^c^Other languages: Arabic, French, Persian, Russian, Serbian. Bold figures: Reflect ORs which are smaller/larger than 1 with a confidence level of 95 %

The odds of asylum-seekers in the medium SSS category being admitted to hospital was 3.18 times [1.06, 9.59] the odds of those in the low SSS category. The association was positive but not significant when comparing asylum-seekers with high SSS with those in the low SSS group (OR = 1.6 [0.49,5.23]) (Table 5).

**Table 5:** Crude and adjusted regression estimates for the association between hospital admissions and SES or need variables among asylum seekers

Explanatory variables		Bivariate models	Multiple regression models (adjusted for sex, age and general health)		
OR [95 % CI]			Model I	Model II	Model III
			Education	SSS	Education + SSS
Education	None/Primary (ref.)	1 *(Ref.)*	1 *(Ref.)*	-	1 *(Ref.)*
	Secondary	1.21 [0.48,3.03]	1.61 [0.51,5.09]	-	1.98 [0.53,7.46]
	Tertiary	1.02 [0.39,2.69]	1.87 [0.57,6.11]	-	1.83 [0.48,6.95]
SSS^a^	Lower (ref.)	1 *(Ref.)*	-	1 *(Ref.)*	1 *(Ref.)*
	Middle	**3.18 [1.06,9.59]**	-	2.6 [0.80,8.40]	2.56 [0.78,8.41]
	High	1.6 [0.49,5.23]	-	1.27 [0.34,4.82]	1.26 [0.33,4.86]
Sex	Male (ref.)	1 *(Ref.)*	1 *(Ref.)*	1 *(Ref.)*	1 *(Ref.)*
	Female	1.7 [0.72,4.01]	2.9 [0.98,8.58]	**3.27 [1.02,10.51]**	**4.13 [1.17,14.60]**
Language	German	1 *(Ref.)*	-	-	-
	English	0.46 [0.17,1.24]	-	-	-
	Others^c^	0.53 [0.22,1.3]	-	-	-
Age (yrs)		0.98 [0.94,1.03]	0.97 [0.92,1.02]	0.98 [0.93,1.03]	0.97 [0.92,1.02]
General health status^b^	“Good” (ref.)	1 *(Ref.)*	1 *(Ref.)*	1 *(Ref.)*	1 *(Ref.)*
	“Bad”	1.83 [0.86,3.89]	**3.28 [1.19,9.04]**	2.59 [0.90,7.46]	**3.35 [1.02,11.04]**
BIC		-	142.07	122.94	130.13
N		-	106	92	91

*OR* indicates the odds ratio compared to the reference group, *CI* indicates 95 % confidence interval, *Pseudo R* ^*2*^ squared for logistic regression, *N* absolute frequency of participating persons. Variations in N result from missing in single items. All data on utilisation of health care services refer to the past 12 months. ^a^SSS: Subjective social status in Germany on a scale from 1 to 10. Lower: 1–4 points. Middle: 5–6 points. High: 7–10 points. ^b^“How is your health in general?” “Bad” = fair/bad/very bad. “Good” = good/very good. ^c^Other languages: Arabic, French, Persian, Russian, Serbian. Bold figures: Reflect ORs which are smaller/larger than 1 with a confidence level of 95 %

In unadjusted models, the odds of reporting utilisation of any physician, GPs, psychotherapists, hospital admissions and unmet medical need in the last 12 months were lower among asylum-seekers who filled out the questionnaire in English relative to those submitting the German version. The direction of the association was inconsistent for ‘Other languages’ relative to ‘German’ (Tables 2, 3, 4, 5 and 6).

**Table 6:** Crude and adjusted regression estimates for the association between unmet medical needs and SES or need variables among asylum seekers

Explanatory variables		Bivariate models	Multiple regression models (adjusted for sex, age and general health)		
OR [95 % CI]			Model I	Model II	Model III
			Education	SSS	Education + SSS
Education	None/Primary (ref.)	1 *(Ref.)*	1 *(Ref.)*	-	1 *(Ref.)*
	Secondary	0.51 [0.21,1.26]	0.44 [0.15,1.25]	-	0.67 [0.21,2.12]
	Tertiary	1.48 [0.65,3.39]	1.42 [0.52,3.85]	-	1.28 [0.44,3.77]
SSS^a^	Lower (ref.)	1 *(Ref.)*	-	1 *(Ref.)*	1 *(Ref.)*
	Middle	0.95 [0.37,2.40]	-	0.92 [0.34,2.51]	0.95 [0.34,2.63]
	High	0.66 [0.25,1.71]	-	0.78 [0.27,2.23]	0.85 [0.29,2.50]
Sex	Male (ref.)	1 *(Ref.)*	1 *(Ref.)*	1 *(Ref.)*	1 *(Ref.)*
	Female	0.57 [0.25,1.29]	0.53 [0.19,1.46]	0.67 [0.23,1.97]	0.65 [0.21,1.98]
Language	German	1 *(Ref.)*	-	-	-
	English	0.5 [0.19,1.28]	-	-	-
	Others^c^	1.01 [0.43,2.35]	-	-	-
Age (yrs)		0.99 [0.95,1.03]	0.99 [0.95,1.04]	0.99 [0.94,1.03]	0.99 [0.94,1.03]
General health status^b^	“Good” (ref.)	1 *(Ref.)*	1 *(Ref.)*	1 *(Ref.)*	1 *(Ref.)*
	“Bad”	**2.11 [1.06,4.18]**	2.13 [0.90,5.04]	2 [0.85,4.71]	2.16 [0.84,5.59]
BIC		-	162.61	149.74	156.5
N		-	105	92	91

*OR* indicates the odds ratio compared to the reference group, *CI* indicates 95 % confidence interval, *Pseudo R* ^*2*^ squared for logistic regression, *N* absolute frequency of participating persons. Variations in N result from missing in single items. All data on utilisation of health care services refer to the past 12 months. ^a^SSS: Subjective social status in Germany on a scale from 1 to 10. Lower: 1–4 points. Middle: 5–6 points. High: 7–10 points. ^b^“How is your health in general?” “Bad” = fair/bad/very bad. “Good” = good/very good. ^c^Other languages: Arabic, French, Persian, Russian, Serbian. Bold figures: Reflect ORs which are smaller/larger than 1 with a confidence level of 95 %

### Adjusted multiple regression estimates

#### Horizontal equity in access to health care: effect of SES adjusted for need

None of the measures of health care access (Tables 2, 3, 4, 5 and 6) were significantly associated with educational attainment or SSS after controlling for age, sex, language and general health status. The significant effect of medium SSS on hospital admissions was mediated to non-significant levels after inclusion of control variables, but the positive direction and magnitude of the association remained (OR = 2.6 [0.80,8.40]) (Table 5). We could observe a statistical trend towards higher odds for hospital admissions and utilisation of psychotherapists for asylum-seekers with medium/high educational attainment and asylum-seekers with medium/high SSS compared to those in the lowest SES category in fully adjusted models (Tables 4 and 5). No such pattern could be observed for the other health care access variables (Tables 2, 3 and 6) in fully adjusted models (I–III).

#### Vertical equity in access to health care: effect of need variables adjusted for SES

Age was significantly associated with utilisation of any type of physician (Table 2) and GPs (Table 3), controlled for the other variables in the models (sex, health status, SSS and/or educational attainment) (I–III). The adjusted effects of age on all other health care access variables were close to 1 and non-significant.

Asylum-seeking women had 3-fold higher odds for hospital admissions compared to men after controlling for health status, age and SSS (OR = 3.27 [1.02,10.51]) (Table 5, Model II). The strength of association between sex and hospital admissions increased (OR = 4.13 [1.17,14.60]) after additional control for educational attainment (Table 5, Model III). There was a trend towards higher odds for utilisation of all other services among women compared to men in all adjusted models (Tables 2, 3 and 4, I–III). An inverse association was observed for unmet medical need: in fully adjusted models women appeared consistently less likely to report any such unmet needs compared to men (Table 6, I–III).

Compared to the reference group, asylum-seekers reporting a ‘Bad’ health status were significantly more likely to visit any type of physician (Table 2), psychotherapists (Table 4) and to be admitted to hospitals (Table 5) after controlling for the other variables in the models (I–III). The adjusted effects of ‘Bad’ health status on utilisation of GPs were positive in direction, statistically not significant and relatively weak in the adjusted models (Table 3, I–III). Utilisation of GPs was thus not strongly associated with health status. The odds of having experienced unmet medical needs (Table 5) among asylum-seekers with ‘Bad’ health status were 2.16 times [0.84, 5.59] the odds of those with ‘Good’ health status after adjustment for age, sex and the two SES variables (Table 6, III).

We observed a trend towards lower odds for utilisation of general practitioners (Table 3) and psychotherapists (Table 4) among those asylum-seekers who filled out the English version of the questionnaire compared to the German-speaking reference group after controlling for all other variables (models I–III).

Additional adjustment for the place of residence (district 1, 2 or 3) did neither change the significance nor the direction of any associations in the calculated models (Tables 2, 3, 4, 5 and 6, I–III) and the place of residence itself had no significant effect on any of the outcome variables (data not shown).

The goodness of fit of the models, as measured by BIC, suggested that for each outcome model II can be considered as the best description of the influence of the variables when compared to model I and III.

## Discussion

This is the first study in Germany to assess equity in access to health care *within* a group of asylum-seekers. We sought to analyse whether the principles of horizontal and vertical equity in health care [2, 15] are met *within* this heterogeneous population group. Our main finding is that access to health care in our study population widely followed the principles of horizontal equity in health care as far as educational attainment is concerned, but showed a social gradient with respect to SSS. After controlling for age, sex, health status, language, and the place of residence, the comprehensive measure of SSS was positively associated with health care access, which we measured by utilisation of four different types of services *and* by unmet medical need. Also, a trend towards higher hospital admissions and use of psychotherapists among asylum-seekers with higher educational attainment remained. This deserves further investigation to assess potential horizontal equities in health care among asylum-seekers. In contrast to populist arguments, no relative overuse of health care was observable among asylum-seekers with low SES.

Utilisation of health services was significantly and positively associated with higher need (worse health status, female sex, higher age) adjusted for SES variables, language and place of residence which indicates that access to health care, measured by utilisation of hospital and specialist services met the principles of vertical equity.

‘Bad’ general health status was consistently the strongest predictor of hospital and specialist service utilisation in the sense that reporting ‘Bad’ general health status was associated with higher odds of hospital and specialist utilisation compared to those reporting ‘Good’ general health status. The adjusted effects ranged from OR = 3.28 [1.19,9.04] for hospital admissions (Table 5) to OR = 5.67 [1.29,24.87] for any type of physician (Table 2), followed by OR = 5.64 [1.41,22.53] for utilisation of psychotherapists (Table 4). In line with the existing literature [24–27], this marks out general health status as an important and strong predictor for health care need among asylum-seekers.

Remarkably, utilisation of general practitioners was not statistically significantly associated with health status, and the regression estimates were comparably weak. Having similar odds for access to primary care despite reporting worse health status, controlled for age, sex and SES, suggests that vertical equity in access to primary care among asylum-seekers could be improved.

Migrant-specific access barriers to comprehensive primary care (e.g., language discordance with GPs) might be exacerbated by legal restrictions which create obstacles to access unless conditions are acute, painful or emergencies. Improving vertical equity in access to primary care could also help to reduce hospital admissions for those with higher needs. However, owing to the lack of information on the reasons for hospitalisation, we cannot conclude whether hospitalisations among our study population were amenable to primary care interventions.

There was a social gradient in reporting unmet medical need across SSS: the higher the SSS, the lower the odds of reporting unmet need (horizontal equity not met). Also, those reporting ‘Bad’ general health status appeared to be more likely to experience unmet needs compared to the reference group (Table 6). This finding underlines the importance to go beyond measures of utilisation when analysing health care access [16]. With respect to unmet medical needs, these findings hint at potential horizontal and vertical inequities in access to care among asylum-seekers and deserve further investigation. Minimising barriers to access to primary care services could be considered as a potential approach to achieve both horizontal and vertical equity in unmet needs among asylum-seekers.

Female gender was consistently the second strongest predictor of health care utilisation, albeit not significant for all types of services. Asylum-seeking women had 4-fold higher odds for hospital admissions compared to men irrespective of age, SES, and place of residence (Table 5, III) and consistently higher odds for utilisation of all other types of services. This gender difference may partly be attributed to higher medical needs among women due to pregnancy and childbirth.

### Strengths and limitations

The main strength of our study is the comprehensive analysis of equity in health care access in its two dimensions: horizontal and vertical equity in realised access (utilisation) and non-access (unmet medical needs). We further deployed a novel approach in measuring SES among asylum-seekers by using a subjective measure of social status (SSS) based on an adaptation of the MacArthur Scale. This allowed us to assess the subjectively perceived positioning of asylum-seeking migrants in the social hierarchy of their “new” environment in Germany. We complemented this subjective measure with an objective measure of educational attainment which allowed a comprehensive assessment of SES in this heterogeneous study population.

Nevertheless, international comparability of SES indicators remains a challenging issue [28] and it is possible that the way we captured educational attainment did not fully reflect the different social implications that are entailed by different degrees from different countries, thus potentially leading to classification errors. Furthermore, there are only very few studies analysing SSS among asylum-seeking or refugee populations [29–31] and further research is necessary to deepen the understanding of the measure in these special migrant populations.

The main limitation of our study is that it was confined to three large administrative districts in a Federal State of Germany without preceding sample size calculation, which may have led to an underpowered sample size for the analyses performed in this study. The administrative districts were conveniently selected from all 44 administrative districts in the State, mainly because collaborations with the local administration could be established in these areas – a ‘*conditio sine qua non’* in terms of accessing the population of asylum-seekers in attempts to conduct a population-based study. In light of the satisfactory response rate, our findings are generalisable to the population of asylum-seekers residing in the three administrative districts, but may not be representative for the Federal State, and are by no means representative for the whole of Germany. While the origins of asylum-seekers in the Federal State of Baden-Württemberg were primarily reported as Syria, Gambia and Eastern Europe [32], the three study districts hosted a large number of asylum-seekers from Iran and Pakistan. Research into health and health care of asylum-seekers is challenged by the lack of (timely) denominator data [33]. This adds to the reason why we cannot make statements on the generalisability regarding the whole Federal State.

Our findings should thus not be blindly inferred to areas beyond the three administrative districts because the parallel health care system created by legal and bureaucratic regulations of the Asylum-Seekers Benefits Act operates in a very context-specific way and may show wide regional variations in the way the regulations are handled, interpreted and implemented by local authorities.

Further limitations are the reliance on self-reported health items, which may imply social desirability, particularly among asylum-seekers whose residence status might at least partially depend on their health status. The self-reported measures of health care utilisation during the last twelve months may gave incurred a recall bias. A systematic bias may lie in the linguistic difficulty of the used indicators to measure access, which may cause a filtering effect in favor of higher educated people. However, about 43 % of all asylum-seekers who participated in our study reported “no degree” (15.7 %) or “primary school” (26.9 %) as highest educational attainment (Table 1), which means that this potential filtering effect may be negligible in our study.

Although each measurement item was translated by two professional translators some questions might not have been fully culturally adapted. Although the survey instruments were deployed in seven languages, some languages could not be covered: we still faced language barriers with many asylum-seekers particularly from Pakistan and Turkey, which was not foreseeable when we prepared for the study.

Finally, approaching asylum-seekers in the frame of their monthly payments led to an under-representation of women in the sample as generally men are picking up the money for the whole family. The gender ratio (male : female) of our sample (2.9 : 1) does not reflect the gender distribution of asylum-seekers in the Federal State (2.0 : 1). This means that asylum-seeking women were underrepresented in our survey, which may have affected our results in terms of health care access for this group.

## Conclusions

This first study of equity in health care access among asylum-seekers in Germany found trends towards horizontal inequities based on SSS for all outcome measures, and on educational attainment for utilisation of psychotherapists and hospitalisations. Utilisation of health care services among asylum-seekers in the study region was in line with the principle of vertical equity. Utilisation of general practitioners was not significantly and only weakly associated with health status. This indicates room for improvement of vertical equity in access to primary care. Unmet medical needs showed a social gradient after controlling for need (horizontal equity not met), and were more likely among those with worse health status, which contributes to the existence of vertical inequities. In light of the exploratory character of the study and the study limitations, we conclude that further confirmatory research is necessary with respect to equity aspects in health care for this population group, especially with respect to potential horizontal and vertical inequities in unmet medical need and primary care. This includes further research into measurement of SES among asylum-seeking and refugee populations.

## Additional files

Additional file 1:
**Supplementary data to methods.** (DOCX 83 kb) Additional file 2:
**Supplementary data to results and logistic regression models.** (DOC 273 kb) Additional file 3:
**Supplementary data to non-responder analysis.** (DOCX 15 kb)
