# Supplementary material for: Equity in access to health care among asylum seekers in Germany: evidence from an exploratory population-based cross-sectional study
Source: BMC Health Serv Res. 2015 Nov 9;15:502. doi: 10.1186/s12913-015-1156-x (PMC4640386; doi:10.1186/s12913-015-1156-x)
Supplement: Additional file 1: — Supplementary data to methods. (DOCX 83 kb) [file 12913_2015_1156_MOESM1_ESM.docx]

**Supplementary File 1**

**Equity in access to health care among asylum seekers in Germany: evidence from an exploratory population-based cross-sectional study**

**Kayvan Bozorgmehr, Christine Schneider, Stefanie Joos**

**Content:**

**Supplementary notes to methods**

1. Development of the questionnaire
2. Translation process
3. Causal diagram of regression models

**Supplementary notes to methods**

**i) Development of the questionnaire**

The concern of this study was to use a questionnaire which is based on the European Health Core Indicators and includes items on health status and access to health care services whilst being in a simple and concise form to be self-completed by asylum seekers. According to a literature review, common measures to assess health status or health care provision are either not tested for validity in refugee populations or are not designed as a self-completion questionnaire (1).

The questionnaire applied in this study comprised questions of European and German health surveys (2, 3) and basic socio-demographic information. Permission to use the items in the questionnaire was obtained. A draft version of the questionnaire was reviewed and modified by professionals who are working with refugees.

**ii) Translation process**

After obtaining information on the principal languages of our study population from the local Welfare Agencies, a German version of the questionnaire was translated into six languages: English, Arabic, French, Persian, Serbian, and Russian.

We used established versions of translated items where such existed. Items which had not yet been translated to the respective languages were translated by two independent certified translators for each language. Discrepancies in translations were resolved by consensus between the two translators.

**iii) Causal diagrams**

1. Hollifield MMD, Warner TDP, Lian NDOM, Krakow BMD, Jenkins JHP, Kesler JMD, et al. Measuring Trauma and Health Status in Refugees: A Critical Review. JAMA. 2002;288(5):611-621.

2. Eurostat (Hg.): Leben in Europa (EU-SILC) - Einkommen und Lebensbedingungen in Deutschland und der Europäischen Union - Fachserie 15 Reihe 3- 2012. Available online. URL: https://<http://www.destatis.de/DE/Publikationen/Thematisch/EinkommenKonsumLebensbedingungen/LebeninEuropa/EinkommenLebensbedingungen.html>.

3. Gößwald A, Lange M, Dölle R, Hölling H. Die erste Welle der Studie zur Gesundheit Erwachsener in Deutschland (DEGS1). Bundesgesundheitsblatt - Gesundheitsforschung - Gesundheitsschutz. 2013;56(5-6):611-619.
