# Supplementary material for: Equity in access to health care among asylum seekers in Germany: evidence from an exploratory population-based cross-sectional study
Source: BMC Health Serv Res. 2015 Nov 9;15:502. doi: 10.1186/s12913-015-1156-x (PMC4640386; doi:10.1186/s12913-015-1156-x)
Supplement: Additional file 2: — Supplementary data to results and logistic regression models. (DOC 273 kb) [file 12913_2015_1156_MOESM2_ESM.doc]

**Supplementary File 2**

**Equity in access to health care among asylum seekers in Germany: evidence from an exploratory population-based cross-sectional study**

**Kayvan Bozorgmehr, Christine Schneider, Stefanie Joos**

# Content:

**Supplementary notes to logistic regression models**

Content: [1](#__RefHeading___Toc420935969)

Table S1: Physician [2](#__RefHeading___Toc420935970)

Table S 1.1: Model 1: Association of education with utilization of physicians (in-and outpatient) [2](#__RefHeading___Toc420935971)

Table S 1.2: Model 2: Association of subjective social status with utilization of physicians (in-and outpatient) [2](#__RefHeading___Toc420935972)

Table S 1.3: Model 3: Association of subjective social status and education with utilization of physicians (in-and outpatient) [3](#__RefHeading___Toc420935973)

Table S2: General practitioner [3](#__RefHeading___Toc420935974)

Table S 2.1: Model 1: Association of education with utilization of general practitioners [3](#__RefHeading___Toc420935975)

Table S 2.2: Model 2: Association of subjective social status with utilization of general practitioners [4](#__RefHeading___Toc420935976)

Table S 2.3: Model 3: Association of subjective social status and education with utilization of general practitioners [4](#__RefHeading___Toc420935977)

Table S3: Psychotherapists [5](#__RefHeading___Toc420935978)

Table S 3.1: Model 1: Association of education with utilization of psychotherapists [5](#__RefHeading___Toc420935979)

Table S 3.2: Model 2: Association of subjective subjective social status (SSS) with utilization of psychotherapists [5](#__RefHeading___Toc420935980)

Table S 3.3: Model 3: Association of subjective social status and education with utilization of psychotherapists [6](#__RefHeading___Toc420935981)

Table S4: Hospital admissions [6](#__RefHeading___Toc420935982)

Table S4.1: Model 1: Association of education with hospital admissions [6](#__RefHeading___Toc420935983)

Table S 4.2: Model 2: Association of subjective social status with hospital admissions [7](#__RefHeading___Toc420935984)

Table S 4.3: Model 3: Association of subjective social status and education with hospital admissions [7](#__RefHeading___Toc420935985)

Table S5: Unmet medical need [8](#__RefHeading___Toc420935986)

Table S 5.1: Model 1: Association of education with unmet medical need [8](#__RefHeading___Toc420935987)

Table S 5.2: Model 2: Association of subjective social status with unmet medical need [8](#__RefHeading___Toc420935988)

Table S 5.3: Model 3: Association of subjective social status and education with unmet medical need [9](#__RefHeading___Toc420935989)

# Table S1: Physician

## Table S 1.1: Model 1: Association of education with utilization of physicians (in-and outpatient)

| **Explanatory variables OR [95%CI]** |  | **Education + gender** | **Education + age** | **Education + general health** | **Education + gender + age** | **Model I final** |
| --- | --- | --- | --- | --- | --- | --- |
| Education | None/ Primary (ref.) | 1 *(Ref.)* | 1 *(Ref.)* | 1 *(Ref.)* | 1 *(Ref.)* | 1 *(Ref.)* |
|  | Secondary | 0.77 [0.27,2.20] | 0.74 [0.19,2.88] | 1.03 [0.34,3.12] | 0.79 [0.20,3.17] | 1.07 [0.24,4.82] |
|  | Tertiary | 0.81 [0.29,2.29] | 0.36 [0.10,1.26] | 1.47 [0.47,4.58] | 0.38 [0.11,1.36] | 0.78 [0.19,3.18] |
| Gender | Male (ref.) | 1 *(Ref.)* |  |  | 1 *(Ref.)* | 1 *(Ref.)* |
|  | Female | 1.22 [0.44,3.40] |  |  | 1.57 [0.38,6.54] | 1.9 [0.41,8.92] |
| Age (yrs) |  |  | **1.16** [1.05,1.29] |  | **1.16** [1.05,1.29] | **1.16** [1.04,1.29] |
| General health status | "Good" (ref.) |  |  | 1 *(Ref.)* |  | 1 *(Ref.)* |
|  | "Bad" |  |  | **6.54** [2.05,20.87] |  | **5.61** [1.32,23.73] |
| Constant |  | **4.41** [2.08,9.33] | 0.11 [0.01,1.57] | 1.98 [0.89,4.43] | 0.09 [0.01,1.43] | 0.05 [0.00,0.92] |
| Wald-Chi |  | 0.51 | 16.16 | 13.18 | 16.56 | 21.97 |
| Pseudo-R-sqr |  | 0.003 | 0.16 | 0.1 | 0.16 | 0.23 |
| Model-df |  | 3 | 3 | 3 | 4 | 5 |
| N |  | 131 | 111 | 129 | 111 | 108 |

| Table S 1.2: Model 2: Association of subjective social status with utilization of physicians (in-and outpatient) |  |
| --- | --- |

| **Explanatory variables OR [95%CI]** |  | **Subjective social status + gender** | **Subjective social status + age** | **Subjective social status + general health** | **Subjective social status + gender + age** | **Model II final** |
| --- | --- | --- | --- | --- | --- | --- |
| Subjective social status | Lower (ref.) | 1 *(Ref.)* | 1 *(Ref.)* | 1 *(Ref.)* | 1 *(Ref.)* | 1 *(Ref.)* |
|  | Middle | 1.51 [0.38,5.90] | 2.54 [0.48,13.42] | 1.65 [0.41,6.69] | 2.52 [0.48,13.34] | 2.55 [0.44,14.84] |
|  | High | 0.53 [0.16,1.71] | 0.81 [0.20,3.24] | 0.63 [0.19,2.12] | 0.8 [0.19,3.30] | 0.98 [0.21,4.55] |
| Gender | Male (ref.) | 1 (Ref.) |  |  | 1 (Ref.) | 1 (Ref.) |
|  | Female | 1.42 [0.41,4.90] |  |  | 2.17 [0.41,11.56] | 2.51 [0.41,15.51] |
| Age (yrs) |  |  | **1.17** [1.04,1.31] |  | **1.17** [1.04,1.32] | **1.19** [1.04,1.37] |
| General health status | "Good" (ref.) |  |  | 1 (Ref.) |  | 1 (Ref.) |
|  | "Bad" |  |  | **4.47** [1.35,14.82] |  | **5.67** [1.29,24.87] |
| Constant |  | 4.74 [1.94,11.58] | 0.06 [0.00,1.70] | 2.62 [0.98,6.99] | 0.04 [0.00,1.49] | 0.01 [0.00,0.89] |
| Wald-Chi |  | 3 | 14.01 | 10.33 | 14.94 | 21.52 |
| Pseudo-R-sqr |  | 0.03 | 0.17 | 0.1 | 0.18 | 0.26 |
| Model-df |  | 3 | 3 | 3 | 4 | 5 |
| N |  | 105 | 96 | 104 | 96 | 94 |

| Table S 1.3: Model 3: Association of subjective social status and education with utilization of physicians (in-and outpatient) |  |
| --- | --- |

| **Explanatory variables OR [95%CI]** |  | **Subjective social status + education + gender** | **Subjective social status + education + age** | **Subjective social status + education + general health** | **Subjective social status + education + gender + age** | **Model III final** |
| --- | --- | --- | --- | --- | --- | --- |
| Subjective social status | Lower (ref.) | 1 *(Ref.)* | 1 *(Ref.)* | 1 *(Ref.)* | 1 *(Ref.)* | 1 *(Ref.)* |
|  | Middle | 1.52 [0.39,6.03] | 3.44 [0.59,20.09] | 1.67 [0.41,6.82] | 3.39 [0.58,19.80] | 2.94 [0.48,18.04] |
|  | High | 0.49 [0.15,1.64] | 0.76 [0.17,3.35] | 0.63 [0.18,2.18] | 0.74 [0.16,3.38] | 0.88 [0.18,4.25] |
| Education | None/ Primary (ref.) | 1 *(Ref.)* | 1 *(Ref.)* | 1 *(Ref.)* | 1 *(Ref.)* | 1 *(Ref.)* |
|  | Secondary | 1.22 [0.31,4.79] | 1.29 [0.22,7.45] | 1.42 [0.35,5.71] | 1.38 [0.23,8.15] | 1.36 [0.22,8.54] |
|  | Tertiary | 0.64 [0.20,2.07] | 0.29 [0.07,1.20] | 1.04 [0.29,3.69] | 0.32 [0.07,1.33] | 0.59 [0.12,2.87] |
| Gender | Male (ref.) | 1 (Ref.) |  |  | 1 (Ref.) | 1 (Ref.) |
|  | Female | 1.48 [0.42,5.27] |  |  | 1.91 [0.35,10.30] | 2.26 [0.37,13.85] |
| Age (yrs) |  |  | **1.196** [1.05,1.36] |  | **1.197** [1.05,1.36] | **1.197** [1.04,1.38] |
| General health status | "Good" (ref.) |  |  | 1 (Ref.) |  | 1 (Ref.) |
|  | "Bad" |  |  | **4.45** [1.27,15.61] |  | 4.3 [0.86,21.35] |
| Constant |  | 5.26 [1.68,16.43] | 0.04 [0.00,1.85] | 2.36 [0.65,8.59] | 0.04 [0.00,1.64] | 0.02 [0.00,1.11] |
| Wald-Chi |  | 3.94 | 17.95 | 10.36 | 18.57 | 22.15 |
| Pseudo-R-sqr |  | 0.04 | 0.22 | 0.11 | 0.22 | 0.27 |
| Model-df |  | 5 | 5 | 5 | 6 | 7 |
| N |  | 104 | 95 | 103 | 95 | 93 |

# Table S2: General practitioner

| Table S 2.1: Model 1: Association of education with utilization of general practitioners |  |
| --- | --- |

| **Explanatory variables OR [95%CI]** |  | **Education + gender** | **Education + age** | **Education + general health** | **Education + gender + age** | **Model I final** |
| --- | --- | --- | --- | --- | --- | --- |
| Education | None/ Primary (ref.) | 1 *(Ref.)* | 1 *(Ref.)* | 1 *(Ref.)* | 1 *(Ref.)* | 1 *(Ref.)* |
|  | Secondary | 0.87 [0.35,2.16] | 0.85 [0.30,2.42] | 1.04 [0.42,2.60] | 0.92 [0.32,2.65] | 1.16 [0.38,3.52] |
|  | Tertiary | 0.59 [0.25,1.40] | 0.37 [0.14,0.99] | 0.79 [0.32,1.93] | 0.38 [0.14,1.05] | 0.49 [0.17,1.39] |
| Gender | Male (ref.) | 1 (Ref.) |  |  | 1 (Ref.) | 1 (Ref.) |
|  | Female | 1.17 [0.51,2.72] |  |  | 1.6 [0.55,4.70] | 2.1 [0.66,6.71] |
| Age (yrs) |  |  | **1.07** [1.02,1.13] |  | **1.07** [1.01,1.13] | 1.06 [1.00,1.12] |
| General health status | "Good" (ref.) |  |  | 1 (Ref.) |  | 1 (Ref.) |
|  | "Bad" |  |  | 2.1 [0.97,4.54] |  | 1.53 [0.62,3.81] |
| Constant |  | 2.23 [1.19,4.19] | 0.39 [0.08,1.96] | 1.49 [0.74,3.01] | 0.35 [0.07,1.83] | 0.33 [0.06,1.87] |
| Wald-Chi |  | 1.67 | 11.72 | 4.59 | 12.48 | 11.62 |
| Pseudo-R-sqr |  | 0.01 | 0.08 | 0.03 | 0.09 | 0.09 |
| Model-df |  | 3 | 3 | 3 | 4 | 5 |
| N |  | 131 | 111 | 129 | 111 | 108 |

| **Explanatory variables OR [95%CI]** |  | **Subjective social status + gender** | **Subjective social status + age** | **Subjective social status + general health** | **Subjective social status + gender + age** | **Model II final** | |  |
| --- | --- | --- | --- | --- | --- | --- | --- | --- |
| Subjective social status | Lower (ref.) | 1 *(Ref.)* | 1 (Ref.) | 1 *(Ref.)* | 1 *(Ref.)* | 1 *(Ref.)* | |  |
|  | Middle | 1.57 [0.58,4.24] | 1.53 [0.51,4.58] | 1.45 [0.54,3.89] | 1.5 [0.50,4.51] | 1.39 [0.46,4.23] | |  |
|  | High | 1.13 [0.42,3.03] | 1.51 [0.50,4.56] | 1.34 [0.49,3.69] | 1.43 [0.47,4.40] | 1.69 [0.53,5.46] | |  |
| Gender | Male (ref.) | 1 (Ref.) |  |  | 1 (Ref.) | 1 (Ref.) | |  |
|  | Female | 1.21 [0.46,3.17] |  |  | 2.12 [0.61,7.40] | 3.04 [0.75,12.33] | |  |
| Age (yrs) |  |  | **1.08** [1.02,1.14] |  | **1.08** [1.01,1.15] | **1.07** [1.01,1.14] | |  |
| General health status | "Good" (ref.) |  |  | 1 (Ref.) |  | 1 (Ref.) | |  |
|  | "Bad" |  |  | 1.71 [0.74,3.92] |  | 1.63 [0.63,4.24] | |  |
| Constant |  | 1.52 [0.77,3.03] | 0.18 [0.02,1.25] | 1.18 [0.53,2.63] | 0.15 [0.02,1.16] | 0.14 [0.02,1.20] | |  |
| Wald-Chi |  | 1.02 | 8.54 | 2.16 | 10.05 | | 10.53 | |
| Pseudo-R-sqr |  | 0.01 | 0.07 | 0.02 | 0.08 | | 0.09 | |
| Model-df |  | 3 | 3 | 3 | 4 | | 5 | |
| N |  | 105 | 96 | 104 | 96 | | 94 | |

| Table S 2.2: Model 2: Association of subjective social status with utilization of general practitioners |  |
| --- | --- |

| Table S 2.3: Model 3: Association of subjective social status and education with utilization of general practitioners |  |
| --- | --- |

| **Explanatory variables OR [95%CI]** |  | **Subjective social status + education + gender** | **Subjective social status + education + age** | **Subjective social status + education + general health** | **Subjective social status + education + gender + age** | **Modell III final** |
| --- | --- | --- | --- | --- | --- | --- |
| Subjective social status | Lower (ref.) | 1 *(Ref.)* | 1 (Ref.) | 1 *(Ref.)* | 1 *(Ref.)* | 1 *(Ref.)* |
|  | Middle | 1.59 [0.58,4.39] | 1.66 [0.53,5.19] | 1.47 [0.54,3.98] | 1.63 [0.52,5.13] | 1.58 [0.50,5.05] |
|  | High | 1.07 [0.39,2.93] | 1.54 [0.49,4.88] | 1.26 [0.45,3.54] | 1.46 [0.45,4.70] | 1.62 [0.48,5.39] |
| Education | None/ Primary (ref.) | 1 (Ref.) | 1 (Ref.) | 1 (Ref.) | 1 (Ref.) | 1 (Ref.) |
|  | Secondary | 0.94 [0.32,2.74] | 0.68 [0.20,2.26] | 0.96 [0.33,2.79] | 0.75 [0.22,2.53] | 0.93 [0.26,3.29] |
|  | Tertiary | 0.51 [0.20,1.33] | 0.34 [0.11,1.00] | 0.6 [0.22,1.63] | 0.36 [0.12,1.07] | 0.39 [0.12,1.28] |
| Gender | Male (ref.) | 1 (Ref.) |  |  | 1 (Ref.) | 1 (Ref.) |
|  | Female | 1.2 [0.44,3.25] |  |  | 1.97 [0.55,7.06] | 2.76 [0.66,11.49] |
| Age (yrs) |  |  | **1.09** [1.02,1.15] |  | **1.09** [1.02,1.16] | **1.08** [1.01,1.15] |
| General health status | "Good" (ref.) |  |  | 1 (Ref.) |  | 1 (Ref.) |
|  | "Bad" |  |  | 1.63 [0.63,4.24] |  | 1.14 [0.40,3.25] |
| Constant |  | 1.91 [0.78,4.66] | 0.21 [0.03,1.65] | 0.14 [0.02,1.20] | 0.18 [0.02,1.53] | 0.18 [0.02,1.68] |
| Wald-Chi |  | 3.29 | 12.69 | 3.27 | 13.86 | 13.45 |
| Pseudo-R-sqr |  | 0.02 | 0.11 | 0.02 | 0.12 | 0.12 |
| Model-df |  | 5 | 5 | 5 | 6 | 7 |
| N |  | 104 | 95 | 103 | 95 | 93 |

# Table S3: Psychotherapists

| Table S 3.1: Model 1: Association of education with utilization of psychotherapists |  |
| --- | --- |

| **Explanatory variables OR [95%CI]** |  | **Education + gender** | **Education + age** | **Education + general health** | **Education + gender + age** | **Model I final** |
| --- | --- | --- | --- | --- | --- | --- |
| Education | None/ Primary (ref.) | 1 *(Ref.)* | 1 *(Ref.)* | 1 *(Ref.)* | 1 (Ref.) | 1 *(Ref.)* |
|  | Secondary | 1.14 [0.39,3.39] | 1.22 [0.40,3.73] | 1.04 [0.33,3.26] | 1.26 [0.40,3.93] | 1.39 [0.39,4.91] |
|  | Tertiary | 0.71 [0.22,2.29] | 0.59 [0.16,2.13] | 0.96 [0.28,3.24] | 0.6 [0.17,2.20] | 1.05 [0.25,4.32] |
| Gender | Male (ref.) | 1 (Ref.) |  |  | 1 (Ref.) | 1 (Ref.) |
|  | Female | 1.05 [0.37,3.00] |  |  | 1.22 [0.38,3.91] | 1.23 [0.32,4.65] |
| Age (yrs) |  |  | 0.96 [0.91,1.02] |  | 0.96 [0.91,1.02] | 0.96 [0.90,1.02] |
| General health status | "Good" (ref.) |  |  | 1 (Ref.) |  | 1 (Ref.) |
|  | "Bad" |  |  | **3.48** [1.23,9.88] |  | **4.32** [1.30,14.40] |
| Constant |  | 0.21 [0.10,0.45] | 0.75 [0.12,4.62] | 0.1 [0.03,0.28] | 0.72 [0.12,4.45] | 0.3 [0.04,2.45] |
| Wald-Chi |  | 0.59 | 3.28 | 6.38 | 3.38 | 8.48 |
| Pseudo-R-sqr |  | 0 | 0.03 | 0.06 | 0.03 | 0.08 |
| Model-df |  | 3 | 3 | 3 | 4 | 5 |
| N |  | 131 | 111 | 129 | 111 | 108 |

| Table S 3.2: Model 2: Association of subjective subjective social status (SSS) with utilization of psychotherapists |  |
| --- | --- |

| **Explanatory variables OR [95%CI]** |  | **Subjective social status + gender** | **Subjective social status + age** | **Subjective social status + general health** | **Subjective social status + gender + age** | **Modell II final** |
| --- | --- | --- | --- | --- | --- | --- |
| Subjective social status | Lower (ref.) | 1 *(Ref.)* | 1 *(Ref.)* | 1 *(Ref.)* | 1 *(Ref.)* | 1 *(Ref.)* |
|  | Middle | 1.01 [0.31,3.28] | 1.05 [0.30,3.74] | 1 [0.30,3.33] | 1.03 [0.29,3.66] | 0.96 [0.26,3.56] |
|  | High | 1.03 [0.32,3.38] | 1.33 [0.39,4.60] | 1.23 [0.34,4.36] | 1.28 [0.37,4.46] | 1.37 [0.36,5.29] |
| Gender | Male (ref.) | 1 (Ref.) |  |  | 1 (Ref.) | 1 (Ref.) |
|  | Female | 1.27 [0.43,3.77] |  |  | 1.46 [0.44,4.79] | 1.53 [0.39,5.94] |
| Age (yrs) |  |  | 0.96 [0.91,1.02] |  | 0.96 [0.91,1.02] | 0.96 [0.90,1.02] |
| General health status | "Good" (ref.) |  |  | 1 (Ref.) |  | 1 (Ref.) |
|  | "Bad" |  |  | **3.94** [1.29,12.06] |  | **5.003** [1.42,17.58] |
| Constant |  | 0.23 [0.10,0.54] | 0.72 [0.10,5.21] | 0.1 [0.03,0.32] | 0.7 [0.10,4.93] | 0.28 [0.03,2.63] |
| Wald-Chi |  | 0.2 | 2.34 | 6.55 | 2.71 | 8.78 |
| Pseudo-R-sqr |  | 0 | 0.02 | 0.06 | 0.03 | 0.1 |
| Model-df |  | 3 | 3 | 3 | 4 | 5 |
| N |  | 105 | 96 | 104 | 96 | 94 |

| Table S 3.3: Model 3: Association of subjective social status and education with utilization of psychotherapists |  |
| --- | --- |

| **Explanatory variables OR [95%CI]** |  | **Subjective social status + education + gender** | **Subjective social status + education + age** | **Subjective social status + education + general health** | **Subjective social status + education + gender + age** | **Model III final** |
| --- | --- | --- | --- | --- | --- | --- |
| Subjective social status | Lower (ref.) | 1 *(Ref.)* | 1 *(Ref.)* | 1 *(Ref.)* | 1 *(Ref.)* | 1 *(Ref.)* |
|  | Middle | 0.97 [0.30,3.16] | 1.06 [0.29,3.79] | 0.96 [0.29,3.21] | 1.04 [0.29,3.76] | 0.93 [0.25,3.47] |
|  | High | 0.94 [0.28,3.11] | 1.2 [0.34,4.22] | 1.2 [0.33,4.34] | 1.16 [0.33,4.10] | 1.29 [0.33,5.04] |
| Education | None/ Primary (ref.) | 1 (Ref.) | 1 (Ref.) | 1 (Ref.) | 1 (Ref.) | 1 (Ref.) |
|  | Secondary | 1.28 [0.39,4.17] | 1.3 [0.39,4.38] | 1.13 [0.32,3.97] | 1.4 [0.40,4.86] | 1.53 [0.38,6.20] |
|  | Tertiary | 0.75 [0.22,2.48] | 0.59 [0.16,2.16] | 1.18 [0.32,4.33] | 0.62 [0.17,2.28] | 1.3 [0.29,5.81] |
| Gender | Male (ref.) | 1 (Ref.) |  |  | 1 (Ref.) | 1 (Ref.) |
|  | Female | 1.32 [0.43,4.01] |  |  | 1.49 [0.44,5.06] | 1.75 [0.41,7.46] |
| Age (yrs) |  |  | 0.96 [0.91,1.02] |  | 0.96 [0.91,1.02] | 0.95 [0.89,1.02] |
| General health status | "Good" (ref.) |  |  | 1 (Ref.) |  | 1 (Ref.) |
|  | "Bad" |  |  | **4.23** [1.30,13.73] |  | **5.64** [1.41,22.53] |
| Constant |  | 0.25 [0.09,0.71] | 0.79 [0.10,5.99] | 0.09 [0.02,0.39] | 0.73 [0.10,5.47] | 0.24 [0.02,2.59] |
| Wald-Chi |  | 0.79 | 3.54 | 6.87 | 3.94 | 9.35 |
| Pseudo-R-sqr |  | 0.01 | 0.04 | 0.07 | 0.04 | 0.1 |
| Model-df |  | 5 | 5 | 5 | 6 | 7 |
| N |  | 104 | 95 | 103 | 95 | 93 |

# Table S4: Hospital admissions

| Table S4.1: Model 1: Association of education with hospital admissions |  |
| --- | --- |

| **Explanatory variables OR [95%CI]** |  | **Education + gender** | **Education + age** | **Education + general health** | **Education + gender + age** | **Model I final** |
| --- | --- | --- | --- | --- | --- | --- |
| Education | None/ Primary (ref.) | 1 *(Ref.)* | 1 *(Ref.)* | 1 *(Ref.)* | 1 *(Ref.)* | 1 *(Ref.)* |
|  | Secondary | 1.29 [0.49,3.40] | 1.18 [0.43,3.27] | 1.22 [0.46,3.21] | 1.38 [0.48,3.97] | 1.61 [0.51,5.09] |
|  | Tertiary | 1.07 [0.40,2.84] | 1.02 [0.36,2.89] | 1.22 [0.45,3.35] | 1.15 [0.39,3.34] | 1.87 [0.57,6.11] |
| Gender | Male (ref.) | 1 (Ref.) |  |  | 1 (Ref.) | 1 (Ref.) |
|  | Female | 1.65 [0.69,3.97] |  |  | 2.54 [0.95,6.79] | 2.9 [0.98,8.58] |
| Age (yrs) |  |  | 0.98 [0.94,1.02] |  | 0.98 [0.93,1.02] | 0.97 [0.92,1.02] |
| General health status | "Good" (ref.) |  |  | 1 (Ref.) |  | 1 (Ref.) |
|  | "Bad" |  |  | 2.16 [0.94,4.95] |  | **3.28** [1.19,9.04] |
| Constant |  | 0.27 [0.14,0.54] | 0.65 [0.14,2.97] | 0.21 [0.09,0.48] | 0.53 [0.12,2.44] | 0.26 [0.05,1.46] |
| Wald-Chi |  | 1.35 | 0.99 | 3.43 | 4.38 | 8.31 |
| Pseudo-R-sqr |  | 0.01 | 0.01 | 0.02 | 0.03 | 0.07 |
| Model-df |  | 3 | 3 | 3 | 4 | 5 |
| N |  | 129 | 109 | 127 | 109 | 106 |

| **Explanatory variables OR [95%CI]** |  | **Subjective social status + gender** | **Subjective social status + age** | **Subjective social status + general health** | **Subjective social status + gender + age** | **Model II final** |
| --- | --- | --- | --- | --- | --- | --- |
| Subjective social status | Lower (ref.) | 1 *(Ref.)* | 1 (Ref.) | 1 *(Ref.)* | 1 (Ref.) | 1 *(Ref.)* |
|  | Middle | 2.83 [0.92,8.67] | 2.82 [0.90,8.81] | **3.22** [1.06,9.75] | 2.66 [0.83,8.53] | 2.6 [0.80,8.40] |
|  | High | 1.53 [0.46,5.09] | 1.51 [0.44,5.19] | 1.64 [0.48,5.63] | 1.34 [0.38,4.76] | 1.27 [0.34,4.82] |
| Gender | Male (ref.) | 1 (Ref.) |  |  | 1 (Ref.) | 1 (Ref.) |
|  | Female | 1.79 [0.66,4.84] |  |  | **3.05** [1.03,9.00] | **3.27** [1.02,10.51] |
| Age (yrs) |  |  | 0.98 [0.94,1.03] |  | 0.98 [0.93,1.02] | 0.98 [0.93,1.03] |
| General health status | "Good" (ref.) |  |  | 1 (Ref.) |  | 1 (Ref.) |
|  | "Bad" |  |  | 1.57 [0.63,3.95] |  | 2.59 [0.90,7.46] |
| Constant |  | 0.18 [0.07,0.44] | 0.38 [0.07,2.03] | 0.15 [0.05,0.44] | 0.37 [0.07,1.98] | 0.2 [0.03,1.28] |
| Wald-Chi |  | 5.17 | 3.99 | 5.69 | 8 | 9.8 |
| Pseudo-R-sqr |  | 0.04 | 0.04 | 0.05 | 0.07 | 0.09 |
| Model-df |  | 3 | 3 | 3 | 4 | 5 |
| N |  | 103 | 94 | 102 | 94 | 92 |

| Table S 4.2: Model 2: Association of subjective social status with hospital admissions |  |
| --- | --- |

| Table S 4.3: Model 3: Association of subjective social status and education with hospital admissions | | | | | |  |
| --- | --- | --- | --- | --- | --- | --- |
| **Explanatory variables OR [95%CI]** |  | **Subjective social status + education + gender** | **Subjective social status + education +  age** | **Subjective social status + education + general health** | **Subjective social status + education + gender + age** | **Model III final** |
| Subjective social status | Lower (ref.) | 1 *(Ref.)* | 1 (Ref.) | 1 (Ref.) | 1 *(Ref.)* | 1 *(Ref.)* |
|  | Middle | 2.81 [0.91,8.73] | 2.79 [0.88,8.77] | **3.16** [1.03,9.68] | 2.68 [0.82,8.71] | 2.56 [0.78,8.41] |
|  | High | 1.42 [0.42,4.86] | 1.4 [0.40,4.89] | 1.59 [0.45,5.61] | 1.24 [0.34,4.50] | 1.26 [0.33,4.86] |
| Education | None/ Primary (ref.) | 1 (Ref.) | 1 (Ref.) | 1 (Ref.) | 1 (Ref.) | 1 (Ref.) |
|  | Secondary | 1.8 [0.58,5.58] | 1.38 [0.43,4.36] | 1.59 [0.52,4.84] | 1.74 [0.52,5.81] | 1.98 [0.53,7.46] |
|  | Tertiary | 1.07 [0.35,3.31] | 0.95 [0.31,2.95] | 1.14 [0.35,3.70] | 1.07 [0.33,3.46] | 1.83 [0.48,6.95] |
| Gender | Male (ref.) | 1 (Ref.) |  |  | 1 (Ref.) | 1 (Ref.) |
|  | Female | 1.99 [0.71,5.60] |  |  | **3.31** [1.08,10.10] | **4.13** [1.17,14.60] |
| Age (yrs) |  |  | 0.98 [0.94,1.03] |  | 0.97 [0.93,1.02] | 0.97 [0.92,1.02] |
| General health status | "Good" (ref.) |  |  | 1 (Ref.) |  | 1 (Ref.) |
|  | "Bad" |  |  | 1.68 [0.64,4.42] |  | **3.35** [1.02,11.04] |
| Constant |  | 0.15 [0.05,0.46] | 0.38 [0.06,2.20] | 0.13 [0.04,0.48] | 0.34 [0.06,2.00] | 0.14 [0.02,1.06] |
| Wald-Chi |  | 5.99 | 4.15 | 6.18 | 8.57 | 10.96 |
| Pseudo-R-sqr |  | 0.05 | 0.04 | 0.05 | 0.08 | 0.1 |
| Model-df |  | 5 | 5 | 5 | 6 | 7 |
| N |  | 102 | 93 | 101 | 93 | 91 |

# Table S5: Unmet medical need

| Table S 5.1: Model 1: Association of education with unmet medical need |  |
| --- | --- |

| **Explanatory variables OR [95%CI]** |  | **Education + gender** | **Education + age** | **Education + general health** | **Education + gender + age** | **Model I final** |
| --- | --- | --- | --- | --- | --- | --- |
| Education | None/ Primary (ref.) | 1 *(Ref.)* | 1 (Ref.) | 1 *(Ref.)* | 1 *(Ref.)* | 1 *(Ref.)* |
|  | Secondary | 0.36 [0.14,0.96] | 0.45 [0.17,1.23] | 0.53 [0.20,1.36] | 0.41 [0.15,1.13] | 0.44 [0.15,1.25] |
|  | Tertiary | 1.42 [0.61,3.28] | 1.13 [0.46,2.76] | 1.97 [0.81,4.80] | 1.06 [0.42,2.63] | 1.42 [0.52,3.85] |
| Gender | Male (ref.) | 1 (Ref.) |  |  | 1 (Ref.) | 1 (Ref.) |
|  | Female | 0.5 [0.22,1.18] |  |  | 0.5 [0.19,1.31] | 0.53 [0.19,1.46] |
| Age (yrs) |  |  | 0.99 [0.95,1.03] |  | 0.99 [0.95,1.03] | 0.99 [0.95,1.04] |
| General health status | "Good" (ref.) |  |  | 1 (Ref.) |  | 1 (Ref.) |
|  | "Bad" |  |  | **2.21** [1.02,4.76] |  | 2.13 [0.90,5.04] |
| Constant |  | 0.94 [0.52,1.70] | 1.2 [0.32,4.53] | 0.46 [0.23,0.96] | 1.37 [0.35,5.36] | 0.83 [0.18,3.76] |
| Wald-Chi |  | 9.33 | 3.76 | 9.91 | 5.84 | 8.72 |
| Pseudo-R-sqr |  | 0.05 | 0.03 | 0.06 | 0.04 | 0.06 |
| Model-df |  | 3 | 3 | 3 | 4 | 5 |
| N |  | 128 | 108 | 125 | 108 | 105 |

| Table S 5.2: Model 2: Association of subjective social status with unmet medical need |  |
| --- | --- |

| **Explanatory variables OR [95%CI]** |  | **Subjective social status + gender** | **Subjective social status + age** | **Subjective social status + general health** | **Subjective social status + gender + age** | **Model II final** |
| --- | --- | --- | --- | --- | --- | --- |
| Subjective social status | Lower (ref.) | 1 *(Ref.)* | 1 *(Ref.)* | 1 *(Ref.)* | 1 (Ref.) | 1 (Ref.) |
|  | Middle | 0.93 [0.36,2.38] | 0.94 [0.36,2.51] | 0.94 [0.37,2.41] | 0.99 [0.37,2.66] | 0.92 [0.34,2.51] |
|  | High | 0.65 [0.24,1.73] | 0.64 [0.23,1.77] | 0.73 [0.27,1.98] | 0.68 [0.24,1.91] | 0.78 [0.27,2.23] |
| Gender | Male (ref.) | 1 (Ref.) |  |  | 1 (Ref.) | 1 (Ref.) |
|  | Female | 0.64 [0.25,1.63] |  |  | 0.58 [0.21,1.64] | 0.67 [0.23,1.97] |
| Age (yrs) |  |  | 0.98 [0.95,1.02] |  | 0.99 [0.95,1.03] | 0.99 [0.94,1.03] |
| General health status | "Good" (ref.) |  |  | 1 (Ref.) |  | 1 (Ref.) |
|  | "Bad" |  |  | 1.83 [0.82,4.06] |  | 2 [0.85,4.71] |
| Constant |  | 1.02 [0.51,2.02] | 1.56 [0.37,6.54] | 0.68 [0.30,1.51] | 1.57 [0.37,6.69] | 1.04 [0.21,5.13] |
| Wald-Chi |  | 2.03 | 1.43 | 2.97 | 2.51 | 4.23 |
| Pseudo-R-sqr |  | 0.01 | 0.01 | 0.02 | 0.02 | 0.03 |
| Model-df |  | 3 | 3 | 3 | 4 | 5 |
| N |  | 103 | 94 | 102 | 94 | 92 |

| Table S 5.3: Model 3: Association of subjective social status and education with unmet medical need |  |
| --- | --- |

| **Explanatory variables OR [95%CI]** |  | **Subjective social status + education + gender** | **Subjective social status + education + age** | **Subjective social status + education + general health** | **Subjective social status + education + gender + age** | **Model III final** |
| --- | --- | --- | --- | --- | --- | --- |
| Subjective social status | Lower (ref.) | 1 *(Ref.)* | 1 *(Ref.)* | 1 *(Ref.)* | 1 *(Ref.)* | 1 *(Ref.)* |
|  | Middle | 0.97 [0.37,2.54] | 0.98 [0.36,2.65] | 1.01 [0.39,2.63] | 1.02 [0.38,2.79] | 0.95 [0.34,2.63] |
|  | High | 0.71 [0.26,1.96] | 0.69 [0.25,1.94] | 0.82 [0.29,2.28] | 0.73 [0.26,2.09] | 0.85 [0.29,2.50] |
| Education | None/ Primary (ref.) | 1 (Ref.) | 1 (Ref.) | 1 (Ref.) | 1 (Ref.) | 1 (Ref.) |
|  | Secondary | 0.55 [0.19,1.59] | 0.68 [0.23,1.98] | 0.77 [0.27,2.16] | 0.61 [0.21,1.83] | 0.67 [0.21,2.12] |
|  | Tertiary | 1.05 [0.41,2.66] | 0.99 [0.38,2.58] | 1.38 [0.51,3.71] | 0.94 [0.36,2.48] | 1.28 [0.44,3.77] |
| Gender | Male (ref.) | 1 (Ref.) |  |  | 1 (Ref.) | 1 (Ref.) |
|  | Female | 0.59 [0.23,1.54] |  |  | 0.55 [0.19,1.59] | 0.65 [0.21,1.98] |
| Age (yrs) |  |  | 0.99 [0.95,1.02] |  | 0.99 [0.95,1.03] | 0.99 [0.94,1.03] |
| General health status | "Good" (ref.) |  |  | 1 (Ref.) |  | 1 (Ref.) |
|  | "Bad" |  |  | 1.95 [0.83,4.58] |  | 2.16 [0.84,5.59] |
| Constant |  | 1.1 [0.46,2.63] | 1.58 [0.35,7.17] | 0.59 [0.21,1.66] | 1.67 [0.36,7.76] | 0.94 [0.17,5.20] |
| Wald-Chi |  | 3.32 | 1.87 | 3.62 | 3.11 | 4.84 |
| Pseudo-R-sqr |  | 0.02 | 0.01 | 0.03 | 0.02 | 0.04 |
| Model-df |  | 5 | 5 | 5 | 6 | 7 |
| N |  | 102 | 93 | 101 | 93 | 91 |
