# Supplementary material for: Equity in access to health care among asylum seekers in Germany: evidence from an exploratory population-based cross-sectional study
Source: BMC Health Serv Res. 2015 Nov 9;15:502. doi: 10.1186/s12913-015-1156-x (PMC4640386; doi:10.1186/s12913-015-1156-x)
Supplement: Additional file 3: — Supplementary data to non-responder analysis. (DOCX 15 kb) [file 12913_2015_1156_MOESM3_ESM.docx]

**Supplementary File 3**

**Equity in access to health care among asylum seekers in Germany: evidence from an exploratory population-based cross-sectional study**

**Kayvan Bozorgmehr, Christine Schneider, Stefanie Joos**

**Content:**

**Supplementary results**

Table S.1: Description of participation and non-response by county

Table S.2: Reasons for Non-Response

Table S.3: Comparison of participating asylum seekers and non-responders

**Supplementary results**

**Table S.1: Description of participation and non-response by county**

|  | **County 1** | **County 2** | **County 3** |
| --- | --- | --- | --- |
|  | Freq. (col%) of AS | | |
| Registered (Total) | 350 (100) | 358 (100) | 309 (100) |
| Direct refusal of participation^a^ | 41 (11.7) | 33 (9.2) | 78 (25.2) |
| Non-response ^b^ | 79 (22.6) | 146 (40.8) | 81 (26.2) |
| Participation in survey | 61 (17.4) | 41 (11.5) | 54 (17.5) |
| Not contacted | 169 (48.3) | 138 (38.5) | 96 (31.1) |

Figures are absolute frequencies and column percents of asylum seekers. ^a^ Reasons for refusal are listed in table S.6. ^b^ Asylum seekers who never sent back the questionnaire without specifying reasons.

**Table S.2: Reasons for refusal**

|  | **Freq. (col%)** |
| --- | --- |
| **Reason of Non-Response** |  |
| Language barriers | 68 (44.4) |
| No interest | 35 (22.9) |
| Other reason | 23 (15) |
| Reason not specified | 16 (10.5) |
| No time | 11 (7.2) |
| Total | 153 (100) |

**Table S.3: Comparison of participating asylum seekers and non-responders**

|  |  | **Responder** | **Non-Responder** | **Chi2** | **p-Value** |
| --- | --- | --- | --- | --- | --- |
|  |  | **Freq. (col%)** | |  |  |
| **Gender** | Male | 101 (64.7) | 108 (70.6) | 0.37 | 0.542 |
|  | Female | 35 (22.4) | 44 (28.8) |  |  |
|  | Not specified | 20 (12.8) | 0 (0) |  |  |
| **Place of residence** | County 1 | 61 (39.1) | 42 (27.5) | 8.705 | 0.0128 |
|  | County 2 | 41 (26.3) | 33 (21.6) |  |  |
|  | County 3 | 54 (34.6) | 78 (51) |  |  |
|  | N (%) | 156 (100) | 153 (100) |  |  |
| **Language** | Arabic | 15 (9.6) | 5 (3.3) | 18.903 | 0.004 |
|  | German | 33 (21.2) | 28 (18.3) |  |  |
|  | English | 48 (30.8) | 9 (5.9) |  |  |
|  | French | 5 (3.2) | 0 (0) |  |  |
|  | Persian | 24 (15.4) | 5 (3.3) |  |  |
|  | Russian | 8 (5.1) | 2 (1.3) |  |  |
|  | Serbian | 23 (14.7) | 13 (8.5) |  |  |
|  | Other | 0 (0) | 91(59.5) |  |  |
|  | N (%) | 156 (100) | 153 (100) |  |  |
